# Improving the performance of community health workers in humanitarian emergencies: a realist evaluation protocol for the PIECES programme

**DOI:** 10.1136/bmjopen-2016-011753

**Published:** 2016-08-16

**Authors:** Brynne Gilmore, Ben Jack Adams, Alex Bartoloni, Bana Alhaydar, Eilish McAuliffe, Joanna Raven, Miriam Taegtmeyer, Frédérique Vallières

**Affiliations:** 1 Centre for Global Health, Trinity College Dublin, Dublin, Ireland; 2 International Medical Corps, Santa Monica, California, USA; 3 International Medical Corps, Turkey; 4 School of Health Systems, Nursing and Midwifery, University College Dublin, Dublin, Ireland; 5 Department of International Public Health, Liverpool School of Tropical Medicine, Liverpool, UK; 6 School of Psychology, Trinity College Dublin, Dublin, Ireland

**Keywords:** Community Health Workers, Humanitarian Emergencies, Realist Evaluation, Health Worker Performance

## Abstract

**Introduction:**

Understanding what enhances the motivation and performance of community health workers (CHWs) in humanitarian emergencies represents a key research gap within the field of human resources for health. This paper presents the research protocol for the Performance ImprovEment of CHWs in Emergency Settings (PIECES) research programme. Enhancing Learning and Research in Humanitarian Action (ELRHA) funded the development of this protocol as part of their Health in Humanitarian Crises (R2HC) call (No.19839). PIECES aims to understand what factors improve the performance of CHWs in level III humanitarian emergencies.

**Methods and analysis:**

The suggested protocol uses a realist evaluation with multiple cases across the 3 country sites: Turkey, Iraq and Lebanon. Working with International Medical Corps (IMC), an initial programme theory was elicited through literature and document reviews, semistructured interviews and focus groups with IMC programme managers and CHWs. Based on this initial theory, this protocol proposes a combination of semistructured interviews, life histories and critical incident narratives, surveys and latent variable modelling of key constructs to explain how contextual factors work to trigger mechanisms for specific outcomes relating to IMC's 300+ CHWs' performance. Participants will also include programme staff, CHWs and programme beneficiaries. Realist approaches will be used to better understand ‘what works, for whom and under what conditions’ for improving CHW performance within humanitarian contexts.

**Ethics and dissemination:**

Trinity College Dublin's Health Policy and Management/Centre for Global Health Research Ethics Committee gave ethical approval for the protocol development phase. For the full research project, additional ethical approval will be sought from: Université St. Joseph (Lebanon), the Ethics Committee of the Ministry of Health in Baghdad (Iraq) and the Middle East Technical University (Turkey). Dissemination activities will involve a mixture of research feedback, policy briefs, guidelines and recommendations, as well as open source academic articles.

## Introduction

### Community health workers

Community health workers (CHWs) are unpaid or paid lay health workers, with a varied range of training, experience and scope of practice.^1^ Often employed to mitigate against the ongoing human resource for health (HRH) crisis,^2–4^ CHWs provide essential primary care at the household and community level. While the training received and roles performed by CHWs differ across contexts, their purpose within local healthcare systems is universal:^5^ to improve the delivery and extend the reach of primary healthcare services in a cost-effective and equitable manner. More often used in low-income and middle-income countries (LMICs), governments and humanitarian organisations alike implement CHW programmes to increase access to care for marginalised populations and to bridge communities with facility-based services.^3^

It is well established that CHWs can make a positive impact on the health and well-being of the communities they serve^6^ and an extensive body of literature supports their effectiveness in the delivery of primary healthcare programmes.^7–9^ Specifically, there is a large body of work on CHWs for maternal and child health^10^ ^11^ and HIV and AIDS programming.^12^ ^13^ Recent studies have also drawn attention to the challenges in implementing CHW programmes including ensuring regular and supportive supervision (F Vallières, E McAuliffe, P Hyland, *et al*. Measuring the supervision of community health workers: developing and validating the perceived supervision scale. *PLoS ONE* in review),^14^ sustaining CHW motivation,^15^ high attrition rates^7^ ^16^ ^17^ and optimising CHW performance,^18^ ^19^ all of which are necessary to ensure successful CHW programmes.^20^

The performance of CHWs and how it relates to motivation and programme implementation is not well understood. For this study, performance is considered in terms of the WHO's dimensions of a well-performing workforce whereby staff are available (they are retained and are regularly present) and competent (they are productive and responsive).^21^ ^22^ Kok *et al*^19^ ^23^ ^24^ provide insight into the performance of CHWs in LMICs, and highlight that contextual factors can influence CHW performance. Aligned to this, it is also recommended that a minimum set of standardised skills, which are context-specific and respond to community needs, are central to the performance management of CHWs.^25^ In contrast, ineffective performance is characterised by variable quality in delivery of services, which is thought to have substantial effects on health.^22^

CHW motivation and performance are linked and appear to be determined by a number of inter-related factors^23^ including access to resources, community embeddedness, ongoing training and manageable workloads.^26^ Motivation and interventions that improve motivation and job satisfaction are considered likely determinants of CHW performance.^17^ ^27^ Similarly, ineffective performance has been attributed to a lack of incentives, poor supervision, demotivation and the absence of ongoing training.^5^ ^28^ ^29^ Despite these considerations, human resource management for improving CHW performance in health interventions and programmes remains inadequately understood.^30^ ^31^ While the current literature offers some guidance on *what* factors are involved in determining the performance of CHWs, little is known about *how* these factors interact to influence CHW performance. This is partially due to the methodological challenges of measuring motivation and performance and due to a preference for assessing the effects of an intervention solely on health outcomes.

Currently, there is a paucity of studies rigorously examining the determinants of CHW performance in humanitarian emergencies, where the need for such evidence is pressing. Health services in humanitarian emergencies are frequently non-existent or under pressure because of the ongoing violence and conflict,^32^ yet the needs for healthcare are increased. The impact of humanitarian emergencies on a population's health is severe and exacerbated by increases in food insecurity, population displacement, crowding and poor access to water and sanitation, lack of resistance to infection, the physical and psychological effects of weapons and exposure to violence, and the collapse of basic healthcare services.^33^ The impact of humanitarian emergencies on health workers and service provision is also extensive and includes the destruction of health facilities, infrastructure, frequent and prolonged shortages in drugs and equipment, loss of qualified health staff, and restricted access to healthcare.^34^ Numerous humanitarian organisations have established community health programmes as a means to increase access to health services during and after humanitarian emergencies in a bid to overcome infrastructural weakness, promote healthy behaviours and task-shift primary care to available cadres.^10^ ^35^ Specifically, CHWs in emergency settings are often used to provide essential services under restrictive and sometimes dangerous situations, and have the potential to contribute to the sustainability of health programmes in the postconflict and recovery stages.^36^ Optimising the performance of CHWs in humanitarian emergencies is likely to be critical to achieving good health outcomes across health conditions, age groups and contexts.^37^ ^38^

Challenges in CHW programming have been documented in Afghanistan, whereby CHWs reported difficulties with resource supplies, community recognition and health systems functioning.^39^ They also reported that the social, gender and cultural norms of CHWs can impact on their responsibilities and duties. For example, the authors noted that some CHWs were reluctant to engage in mental health activities, given its stigmatising nature in most contexts. Similarly, health workers operating in Northern Uganda during the conflict faced physical and emotional trauma and other demotivating factors such as insecurity, a disconnect from social systems, and unstable and under-resourced working conditions. Despite these challenges, CHWs continued to demonstrate innovative coping strategies and strong resilience, such as de-identifying themselves as health workers by sleeping in patients’ quarters and not wearing uniforms, finding strength in their faith or turning simple items such as plastic bags into medical supplies.^40^ A better understanding of how to support and motivate CHWs in humanitarian contexts, how to ensure their motivation is sustained and how motivation impacts performance requires methodologies that are (1) reflective of the complexity and variability of CHW programmes and that (2) can respond to the contextual conditions of the environment. Since contextual factors have been found to influence the performance of CHWs in development settings,^41^ understanding what enhances the motivation and performance of CHWs working in humanitarian emergencies represents a key research gap within the field of HRH.

In this paper, we present the protocol of a realist evaluation and describe an initial programme theory (IPT) that aims to explain CHW performance. The research background is presented first, followed by the methodology, which describes how we derived our IPT, followed by an explanation of the planned approach and research design. The protocol ends with a discussion of the methodological issues of the study. Taken together, this protocol aims to describe a realist evaluation that answers the question: What improves performance of CHWs in humanitarian contexts?

## Background

### Intervention and study setting

Starting in September 2016, the proposed research will be carried out over 2 years across three countries (Iraq, Lebanon and Turkey). Since the beginning of the crises in Syria and Iraq, International Medical Corps (IMC) has used CHW interventions to address a shortage in the health workforce, provide access to healthcare for the most hard-to-reach populations and ensure that services are aligned to beneficiary needs. Operating out of field hospitals, primary healthcare clinics and mobile medical units, CHWs are locally recruited from refugee populations to help deliver health education and medical outreach to conflict-affected beneficiaries. In addition to providing CHWs with a stipend and non-financial incentives, IMC also trains CHWs on maternal and child health, chronic non-communicable diseases, child protection and psychosocial support, recognition of diseases prone to outbreak (ie, cholera, measles) and behaviour change communication. Each CHW then serves a population of ∼1000 displaced and conflict-affected persons, providing (1) referrals to IMC-supported services for treatment, (2) delivery of timely and effective health messaging and (3) public health surveillance.

As part of IMC's CHW programmes, over 90 CHWs have been selected from the Syrian refugee population in Southern Turkey to carry out household visits among Syrian refugees in urban areas in the Syrian border cities of Mersin, Reyhanli, Kilis, Nizip and Sanliurfa.

In Iraq, the study will take place in Erbil, Duhok and Ninewa Governorates, in camps and communities among refugees and internally displaced persons (IDPs) displaced from Syria and areas occupied by the Islamic State of Iraq and the Levant (ISIL) as well as in the Baghdad region. In Northern Iraq, a further 90 CHWs work in three formats: from Mobile Medical Units in collective centres in towns and cities, in urban settings serving IDP populations that have fled to the Kurdish Region of Iraq, and in those in formal refugee camps.

In Lebanon, the study will take place in Tripoli, Akkar, Bekaa, Beirut and Mount Lebanon, and the South of Lebanon among the Syrian refugee population. Here, IMC has enlisted more than 100 CHWs to carry out health education across the country, mostly in informal tent settlements. In total, ∼300 CHWs are working across these three countries, directly serving over 300 000 community members across camp and non-camp settings, with refugees and IDPs, and with low-income and middle-income community members.

## Aims and objectives

The aim of this study is to provide evidence that will inform the development of interventions to support and improve the performance of CHWs and improve CHW programmes in humanitarian crises settings. The objectives of this study are (1) to address current knowledge gaps in terms of what enhances the performance of CHWs in humanitarian emergencies, and (2) to contribute to the evidence base for the better design of CHW programmes within humanitarian contexts. This evaluation, while expanding our knowledge of what works to improve CHW performance, will further elucidate the specific needs of CHWs in specific contexts within humanitarian emergencies (refugee vs IDP camps, urban vs rural non-camp settings, etc) and inform the design of strategies that will improve performance, with a view to improving healthcare outcomes for the populations which CHWs serve. The study is timely when a number of scholars are calling for a shift from more traditional empirical studies to ones that consider the complex nature of such interventions and the importance of whole systems thinking.^42–46^

## Methods

This study employs a realist evaluation using multiple cases across purposively selected humanitarian emergency contexts where IMC is currently implementing CHW programmes. The complexity and variability of CHW programmes across settings lends itself particularly well to realist studies, and realist methods have been recently recommended for the study and understanding of CHW motivation and performance.^19^ Realist methods are particularly applicable to humanitarian emergencies, where CHW motivation and performance are likely to show a different pattern.

**Figure 1:**
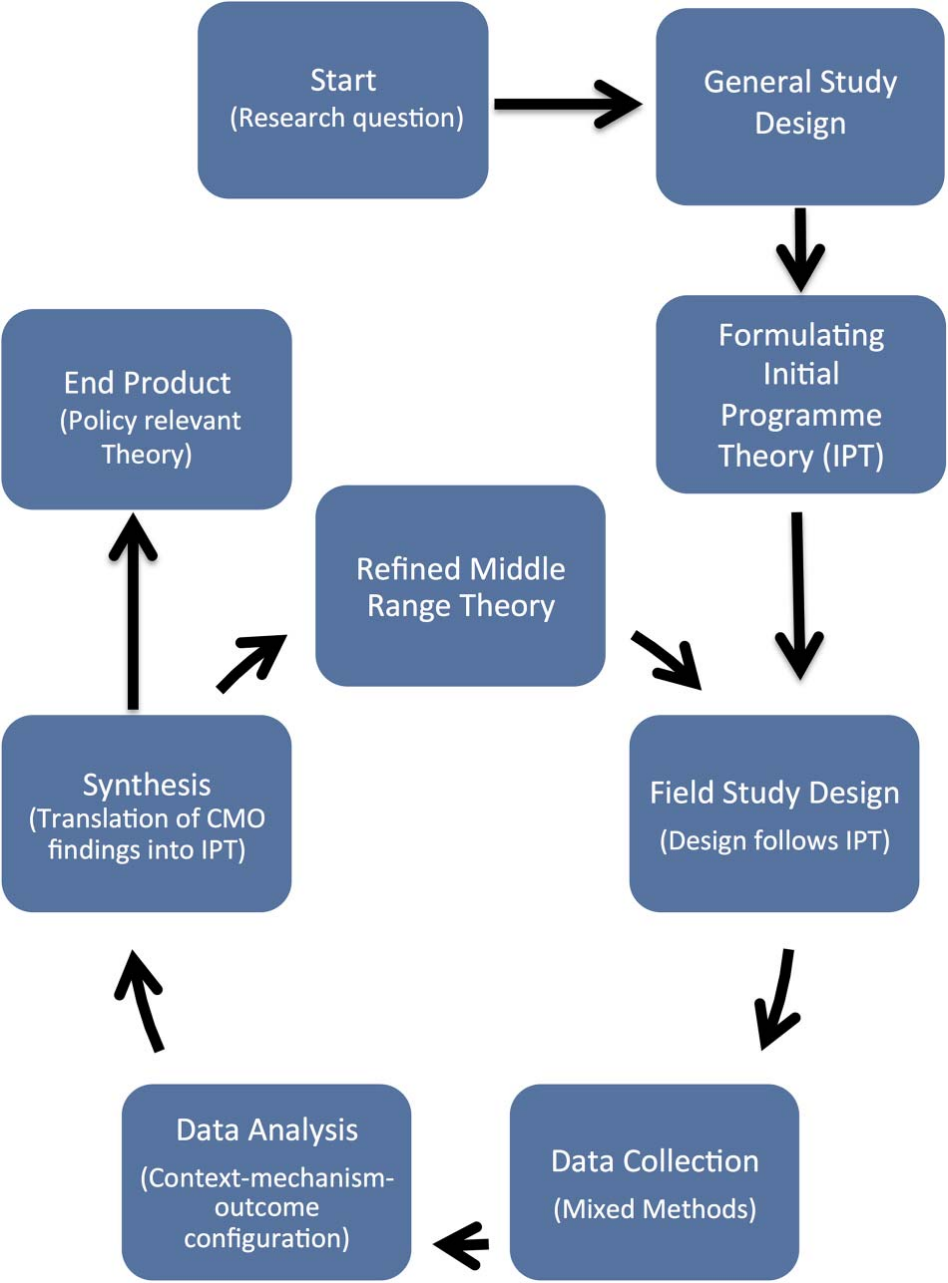
Realist evaluation research cycle (adapted from Van Belle *et al*^47^). CMO, context-mechanism-outcome.

The cycle of a realist evaluation, adapted from Van Belle *et al*,^47^ is outlined in figure 1. Within realist evaluations, initial theories around programmes, or IPTs, are first developed. As realist evaluation sees programmes as theories incarnate, the IPT describes how the programme is expected to work. The IPT is subsequently refined through research conducted across various sites, with the results of this process being more contextually relevant and evidence-based programme theories.^48^ These refined programme theories may in turn be compared to developing a middle-range theory (MRT) on CHW performance in humanitarian emergencies. The MRT produced through this process of programme specification is defined as the “theory that lies between the minor but necessary working hypotheses…and the all-inclusive systematic efforts to develop a unified theory that will explain all the observed uniformities of social behaviour, social organization and social change” (ref. 49, p. 39). The MRT therefore acts as an explanatory framework describing which inputs (ie, components of the IMC intervention) and contextual conditions produce the subsequent mechanisms required to generate change (ie, CHW performance). As stated by Pawson and Tilley,^50^ realist evaluation research follows the ‘traditional research cycle’ of hypothesis (theory) generating and testing. Similar to more traditional types of research, realist evaluations encourage multiple rounds, or iteration in data collection, with each round using previous findings to provide more programme specification.

The methods used in realist studies are informed by the IPT, with realist evaluations themselves being methods neutral.^47^ In the case of this particular study, a combination of quantitative and qualitative methods was selected. Specifically, quantitative methods will be used to (1) measure the various latent constructs described in the IPT using confirmatory factor analysis, (2) ascertain the association between these latent constructs using multiple mediation analysis. Qualitatively, life histories and critical incident reviews with CHWs will be used to understand how these variables influence one another to impact on CHW performance. Research sites were purposefully selected to ensure that they provide sufficient opportunities to test parts of the IPT. Intraprogramme studies (eg, the same programme implemented across different groups) or the same programme being run in different contexts is useful to refine and develop further programme theories and increase the transferability of the findings.^51^

### Formation of IPT

In line with suggestions from Pawson and Sridharan,^52^ the extraction of the IPT for PIECES' protocol was conducted through an in-depth analysis of literature and programme documents, and through interviews with programme developers and managers held between October and December 2015. Additionally, several focus group discussions (FGDs) and semistructured interviews (SSIs) were conducted with CHWs working within IMC's CHW programmes. Table 1 highlights the documentation reviews and methods used to assist in the formation of the IPT. Important to note is that document collection and analysis was done prior to stakeholder input, with context-mechanism-outcome configurations (CMOCs) being developed and further refined through the FGDs and SSIs. The interview guides were developed based on literature/document findings. This process was done to provide contextual information for further refinement and elicitation of the IPT.

**Table 1:** Literature and stakeholder input consulted as part of the IPT development process

Source	Emerging theories and performance factors	Notes
Literature and documentation		
CHW motivation and performance literature	Self determination theory^53^ Measures of burnout^54^ Individual factors Incentives (financial and non-financial) Recognition/respect Work factors (training, support and supervision, recruitment processes) Performance literature and performance outcome monitoring^55^ Trust/relationships (community and health service)	Little information from emergency contexts, majority from development contexts
IMC CHW reports	Outcome (performance) indicators	Outcomes
IMC CHW programme design	Programme design and intervention inputs Outcome (performance) indicators	Context and outcomes
Stakeholder input		
IMC CHW programme architect for Middle East	Work factors (specifically recruitment) Organisational commitment^56^	Continual feedback into IPT
IMC CHW programme managers SSIs (n=5)	Organisational justice^57^ Community commitment Outcome feedback (need to see change) Communication skills	(2) With managers from Turkey (1) With manager from Iraq (2) With managers from Lebanon
CHW focus group discussions (FGD) (n=3)	Organisational justice Psychosocial support/trauma burnout Incentives Recognition/respect Outcome feedback/acknowledgement (need to see change)	(2) With CHWs in Turkey, working in 2 programme sites (Rayhanli and Kilis) (1) With CHWs in Lebanon (from Bekaa and Beirut/Mt. Lebanon)
CHW SSI (n=1)	Incentives Organisational justice Community commitment Psychosocial support/trauma burnout	(1) via Skype with CHW in Erbil, Iraq

CHW, community health worker; IMC, International Medical Corps; IPT, initial programme theory; SSI, semistructured interview.

**Figure 2:**
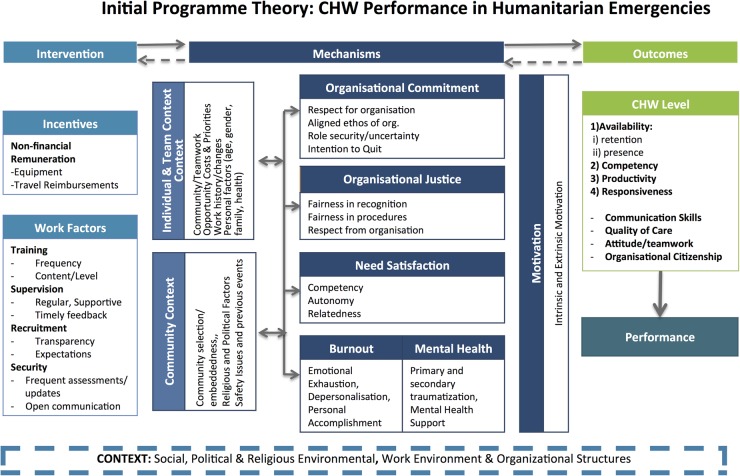
PIECES' initial programme theory. The bidirectional errors depict that change (or producing an outcome) does not necessarily happen in a unilateral direction. The context, mechanisms and outcome all influence one another. CHW, community health worker.

After the completion of IPT extraction, analysis using the CMOC framework as an analytical tool, and the articulation of the associated programme theories was used to create a visual representation.^48^ PIECES' IPT postulates that a CHW programme's inputs should promote organisational commitment, need satisfaction, psychosocial support and a sense of organisational justice, while also mitigating against burnout among CHWs. These factors combine to influence motivation, which acts as a key determinant of CHW performance. The IPT further suggests that improved CHW performance in the short term will lead to improved health outcomes in the long term. Conditions for a good CHW programme include appropriate incentives, strong work factors (ie, CHW training, supervision, recruitment, security) and consideration for the individual and community contexts. Figure 2 visually presents the above description of PIECES' initial theory that was elicited through the process described in table 1.

### Field study design

In line with realist evaluation methodology, our field study design is based on the developed IPT. For this particular study, we will adopt a multiple case study design,^58^ whereby the IPT will be further refined within each case study (ie, Iraq, Lebanon and Turkey) to develop more contextually relevant programme theories of how to enhance CHW performance in humanitarian settings. Case study comparisons can then compare similar CMOCs and produce transferable recommendations across contexts.

#### Methods and tools

Table 2 describes the methods and tools that will be used to explore and refine the concepts that emerged from the IPT. Each process will be completed within all three case studies. First, and aligned to the International Test Commission principles,^59^ existing and, where possible, validated scales measuring the indicators for the mechanisms outlined in the IPT will be translated and back-translated. These indicators for the mechanisms will then be measured at three different time points throughout the proposed 24-month study: baseline (month 3), midterm (month 12) and endline (month 21). Validity will be assessed at baseline using measurement modelling procedures and internal reliability assessed using composite reliability measures. Important to note, however, is that changes to the methods may occur to best refine the programme theories based on findings from the baseline data collection.

**Table 2:** Methods for theory refinement

Concept/theory to be explored	Proposed methods and tools
Intervention inputs	Document reviews KIIs with managers, supervisors and CHWs
CHW performance and outcome	KIIs with managers
CHW performance factors	Life histories and critical incident narratives with CHWs KIIs with managers and supervisors Latent variable modelling
Outcome: quality	Household surveys Semistructured interviews with programme beneficiaries
Outcome: competency	CHW knowledge attitude and practice survey
Outcome: availability, productivity	CHW weekly reporting check cards

CHW**,** community health worker**;** KII**,** key informant interview.

Life histories and critical incident reviews with CHWs will be used to understand their experiences and perceptions relating to performance and their work. These have been used previously for health worker research during conflict and in postconflict settings.^40^ SSIs with programme beneficiaries and key informant interviews (KIIs) with programme staff will be used to explore particular issues of performance that relate to beneficiaries, and provide specialised knowledge on the programme functioning, respectively.

### Sampling

Sampling will be done at the level of the IMC CHW intervention; as such, all participants will be either working with the programme (managers and CHWs) or programme beneficiaries. For all quantitative CHW surveys, the sample of CHWs included will be an estimated 300+ across the three settings, as there are ∼90 CHWs in Turkey, 90 in Iraq and 100 in Lebanon. CHWs will be contacted from current IMC records and all CHWs working for IMC will be invited to partake in the survey. Programme beneficiaries will be selected using convenience sampling methods to participate in the household survey measuring for quality of care. For all qualitative interviews, participants will be selected using purposive sampling. Here, respondents will be deliberately selected on the basis of features or characteristics that will represent a range of stakeholders and enable a detailed understanding of the topic.^60^ CHWs and managers/supervisors will be purposively sampled based on age, gender, place of work and length of time as a CHW in order to obtain the maximum variation in types of experiences. Programme beneficiaries will be selected based on whether they are considered IDPs or refugees, whether they are in a camp or non-camp setting, age, gender and type of services received.

### Data collection

#### Realist techniques

When possible and in line with the methods, data collection will adhere to realist evaluation techniques. Qualitative information from life histories, critical incident narratives, SSIs and (KIIs) will incorporate the realist interview technique,^48^ similar to the ‘teacher–learner’ method. This includes teaching the participant the study team's programme theories, having the participant learn and subsequently teach their own theories regarding the question, accompanied by their own theory refinement. Similarly, all quantitative results will be fed back to CHWs and beneficiaries to stimulate discussion based on the findings. Data in the form of records and documents will also be collected to provide additional contextual information including programme inputs and outputs.

### Analysis

Analysis will first be undertaken at the case study level. Measurement modelling will be used to model the link between the observed measures (items) and their hypothesised underlying factors as identified from the literature (ie, latent constructs). Multiple mediation conditional process analysis will be used to estimate both the total and specific indirect effects, and to contrast different indirect effects.^61^ The total effect of the independent variables on the dependent variable (CHW performance) will therefore be apportioned to (1) the direct effect of the independent variable on the dependent variable and to (2) the indirect effect of the independent variable on multiple mediating variable(s), followed by the effect of the mediating variables on the dependent variable. Multivariate regression will then be applied to explore how changes in independent and mediating variables at midterm predict changes in dependent variables at midterm and endline while controlling for country, type of CHW programme (rural, urban, camp, settings), gender, age, income and education. Findings from the quantitative results will then be further explored through further discussions with CHWs, CHW managers and beneficiaries to complement the development of the CMOCs.

Qualitative data analysis (including document reviews) will use approaches to investigate CMOCs and refine the theory. This will be done by thematically analysing the data, with initial codes being informed deductively by the IPT (codes, eg, ‘burnout’ and ‘organisational commitment’). Using the CMOCs concurrently as an analytical framework will help to identify patterns in the themes, and work inductively to refine the initial theory. Stakeholders (actors) inherently have mechanisms and these, combined with the right contextual factors, produce the generative mechanisms related to outcomes.^62^ It is understanding and explaining this interaction (of mechanisms from individuals and society, combined with contextual factors), which is an outcome of the analysis.

Once the initial analysis of each case is complete, an intraprogramme case study comparison will occur. The IPT will subsequently be refined through each case study, and the comparison of the refined programme theory may help formulate a programme theory that is of middle range. Our refined programme theories will highlight how to enhance CHW performance, by describing the CMOCs, which detail what works (outcome), as well as how (mechanisms) and under what conditions (context). By undergoing this process across three case studies, our programme theories will have a stronger evidence base and therefore be transferable to other CHW programmes within IMC and in other similar contexts.

### Ethics and dissemination

Trinity College Dublin's Health Policy and Management/Centre for Global Health Research Ethics Committee (HPM/CGH REC) gave ethical approval for the protocol development phase of this research. For the full research project, ethical approval will be sought within each research country, in addition to Trinity College Dublin: Université St. Joseph in Lebanon, the Ethics Committee of the Ministry of Health in Baghdad, Iraq and the Middle East Technical University in Turkey. In addition, an early context analysis will inform if other permissions, for example, at camp or community level, are required. Informed consent will be required from all participants prior to data collection.

Three main audience groups have been targeted for dissemination activities: research participants, practitioners (non-governmental organisations) and academics who work in the field of health in humanitarian contexts. Dissemination activities will therefore involve a mixture of research feedback, policy briefs, guidelines and recommendations, as well as open source academic articles. Additionally, built into the research strategy is a social media platform, including a website, formation of a working group and workshop/meeting presentations at relevant international conferences.

## Discussion

Community health researchers are emphasising the need for contextually relevant and explanatory methodologies to provide insight into community health programmes to strengthen programme design.^19^ ^63^ ^64^ Realist evaluations are well placed to address this demand due to its epistemological approach and assumptions of health programmes, with community health research standing to benefit from adapting such methodologies.

The lack of previous studies on CHWs within emergency contexts implies that most of what is presented in the IPT has been largely conjectured from studies of CHWs in LMICs, most of which is less specific to the emergency context literature. This has led to the development of a more ‘generic’ IPT, which may be relevant to the emergency and development contexts. It was, however, also refined though consultation with stakeholders within the programme, with findings having been incorporated into its current form. From these early findings, it is possible that there is some overlap of factors affecting performance of CHWs. However, there were other factors such as CHW safety, camp versus non-camp environments and the need for regular security updates that may be unique to humanitarian emergency contexts. The detailed contextual exploration in the full study phase will work to understand how these factors influence performance, with an emphasis on factors specific to emergency contexts.

This study will provide important information relating to the study question, as well as contribute to the methodological advancement of realist evaluations specifically within humanitarian emergencies. Since there is little precedent to follow in terms of conducting Realist Evaluations (REs) within such environments, the authors are cautious of unforeseen methodological issues that may arise. To this end, important ‘methodology checks’ such as periodical reviews from an outside realist expert, presentations to realist working groups for additional advice and frequent consortium check-ins, have been designed into the protocol, as discussed above. Accordingly, the research team will also be reporting on the use of this methodology in addition to the findings from the study itself. Additionally, working across three complex humanitarian settings may present unforeseen challenges and requires a level of flexibility and/or adaptability of the protocol during the research process. Any alterations to the schedule will be discussed with the research consortium and efforts will be made to maintain the integrity of the overall protocol, with rigour and realist evaluation standards kept as a priority. If the level of flexibility required to continue the research deviates too far from the protocol, the consortium will evaluate the ethical and methodological consequences before agreeing on the best way forward.

## Conclusions

Factors that contribute to health worker performance, including the motivational and contextual factors that create an enabling environment for CHWs to perform effectively, are poorly understood in humanitarian emergencies.^65^ The development of robust, context-informed, evidence-based guidelines for CHW programmes in humanitarian emergencies will therefore help ensure the delivery of high-quality services, while also being reflective of CHW needs. Realist evaluations offer a useful way of doing this due to their flexibility and usefulness within complex interventions and can be adapted flexibly to humanitarian emergencies. To the best of the authors' knowledge, this is the first attempted realist evaluation conducted in an emergency context.

## References

[1] World Health Organisation, Alliance GHW. Economic evaluation of community based practitioners in low-and middle-income countries. Geneva, Switzerland, 2015.

[2] Lewin S, Munabi-Babigumira S, Glenton C Lay health workers in primary and community health care for maternal and child health and the management of infectious diseases. *The Cochrane Library.* Issue 3 2010:209. 10.1002/14651858.CD004015.pub3 PMC6485809 20238326

[3] Gilmore B, McAuliffe E Effectiveness of community health workers delivering preventive interventions for maternal and child health in low- and middle-income countries: a systematic review. BMC Public Health 2013;13:847 10.1186/1471-2458-13-847 24034792 PMC3848754

[4] Hongoro C, McPake B How to bridge the gap in human resources for health. Lancet 2004;364:1451–6. 10.1016/S0140-6736(04)17229-2 15488222

[5] Sato Y, Pongvongsa T, Nonaka D Village health volunteers’ social capital related to their performance in Lao People's Democratic Republic: a cross-sectional study. BMC Health Serv Res 2014;14:123 10.1186/1472-6963-14-123 24620729 PMC3984685

[6] Health Workforce alliance, UNHCR, UNICEF, et al Joint Statement: Scaling up the Community-Based Health Workforce for Emergencies. 2011 http://www.who.int/workforcealliance/knowledge/publications/alliance/jointstatement_chwemergency_en.pdf (accessed August 2016).

[7] Vareilles G, Pommier J, Kane S Understanding the motivation and performance of community health volunteers involved in the delivery of health programmes in Kampala, Uganda: a realist evaluation protocol. BMJ Open 2015;5:e006752 10.1136/bmjopen-2014-006752 PMC4316434 25631314

[8] Lewin S, Munabi-Babigumira S, Glenton C Lay health workers in primary and community health care for maternal and child health and the management of infectious diseases. *The Cochrane Library* 2010. 10.1002/14651858.CD004015.pub3 PMC6485809 20238326

[9] Lassi ZS, Haider BA, Bhutta ZA Community-based intervention packages for reducing maternal and neonatal morbidity and mortality and improving neonatal outcomes. *The Cochrane Library* 2010. 10.1002/14651858.CD007754.pub2 21069697

[10] Bhutta ZA, Lassi ZS, Pariyo G Global experience of community health workers for delivery of health related millennium development goals: a systematic review, country case studies, and recommendations for integration into national health systems. Geneva: World Health Organization, 2010:391.

[11] Brenner JL, Kabakyenga J, Kyomuhangi T Can volunteer community health workers decrease child morbidity and mortality in southwestern Uganda? An impact evaluation. PLoS ONE 2011;6:e27997 10.1371/journal.pone.0027997 22194801 PMC3237430

[12] Mwai GW, Mburu G, Torpey K Role and outcomes of community health workers in HIV care in sub-Saharan Africa: a systematic review. J Int AIDS Soc 2013;16:18586. 24029015 10.7448/IAS.16.1.18586 PMC3772323

[13] Hermann K, Van Damme W, Pariyo GW Community health workers for ART in sub-Saharan Africa: learning from experience—capitalizing on new opportunities. Hum Resour Health 2009;7:31 10.1186/1478-4491-7-31 19358701 PMC2672918

[14] Roberton T, Applegate J, Lefevre AE Initial experiences and innovations in supervising community health workers for maternal, newborn, and child health in Morogoro region, Tanzania. Hum Resour Health 2015;13:19 10.1186/s12960-015-0010-x 25880459 PMC4403773

[15] Banek K, Nankabirwa J, Maiteki-Sebuguzi C Community case management of malaria: exploring support, capacity and motivation of community medicine distributors in Uganda. Health Policy Plan 2015:30:451–61. 10.1093/heapol/czu033 24816572 PMC4385822

[16] Marchal B, De Brouwere V Global human resources crisis. Lancet 2004;363:2191–2. 10.1016/S0140-6736(04)16518-5 15220050

[17] Chen L, Evans T, Anand S Human resources for health: overcoming the crisis. Lancet 2004;364:1984–90. 15567015 10.1016/S0140-6736(04)17482-5

[18] Naimoli JF, Frymus DE, Wuliji T A community health worker “logic model”: towards a theory of enhanced performance in low-and middle-income countries. Hum Resour Health 2014;12:56 10.1186/1478-4491-12-56 25278012 PMC4194417

[19] Kok MC Performance of Community Health Workers: optimizing the benefits of their unique position between communities and the health sector. 2015 https://www.kit.nl/health/wp-content/uploads/publications/5641fbb74cc7f_Kok%202015%20Performance%20of%20CHWs.pdf (accessed August 2016).

[20] Strachan DL, Källander K, ten Asbroek AHA Interventions to improve motivation and retention of community health workers delivering Integrated Community Case Management (iCCM): stakeholder perceptions and priorities. Am J Trop Med Hyg 2012;87(5 Suppl):111–19. 10.4269/ajtmh.2012.12-0030 23136286 PMC3748511

[21] WHO. *World health report—working together for health* 2006.

[22] Haines A, Sanders D, Lehmann U Achieving child survival goals: potential contribution of community health workers. Lancet 2007;369:2121–31. 10.1016/S0140-6736(07)60325-0 17586307

[23] Kok MC, Dieleman M, Taegtmeyer M Which intervention design factors influence performance of community health workers in low- and middle-income countries? A systematic review. Health Policy Plan 2015;30:1207–27. 10.1093/heapol/czu126 25500559 PMC4597042

[24] Kok MC, Dieleman M, Taegtmeyer M Which intervention design factors influence performance of community health workers in low-and middle-income countries? A systematic review. Health Policy Plan 2015;30:1207–27. 10.1093/heapol/czu126 25500559 PMC4597042

[25] Global Health Workforce Alliance, WHO. Integrating community health workers in national health workforce plans. Geneva, Switzerland, 2010.

[26] Raven J, Akweongo P, Baba A Using a human resource management approach to support community health workers: experiences from five African countries. Hum Resour Health 2015;13:45 10.1186/s12960-015-0034-2 26324423 PMC4556018

[27] Rowe AK, de Savigny D, Lanata CF How can we achieve and maintain high-quality performance of health workers in low-resource settings? Lancet 2005;366:17–23. 10.1016/S0140-6736(05)67028-6 16168785

[28] Kelly JM, Osamba B, Garg RM Community health worker performance in the management of multiple childhood illnesses: Siaya District, Kenya, 1997–2001. Am J Public Health 2001;91:1617–24. 10.2105/AJPH.91.10.1617 11574324 PMC1446843

[29] AbouZahr C, Gollogly L, Stevens G Better data needed: everyone agrees, but no one wants to pay. Lancet 2010;375:619–21. 10.1016/S0140-6736(09)60004-0 19150130

[30] Jaskiewicz W, Tulenko K Increasing community health worker productivity and effectiveness: a review of the influence of the work environment. Hum Resour Health 2012;10:38 10.1186/1478-4491-10-38 23017131 PMC3472248

[31] Dieleman M, Harnmeijer JW Improving health worker performance: in search of promising practices. Geneva: World Health Organization, 2006:5–34.

[32] Janneck L, Cooper N, Frehywot S Human resources in humanitarian health working group report. Prehosp Disaster Med 2009;24(Suppl 2):s184–93. 19806538 10.1017/s1049023x00021567

[33] Banatvala N, Zwi AB Conflict and health. Public health and humanitarian interventions: developing the evidence base. BMJ 2000;321:101–5. 10884265 10.1136/bmj.321.7253.101 PMC1127723

[34] Footer KH, Meyer S, Sherman SG On the frontline of eastern Burma's chronic conflict—listening to the voices of local health workers. Soc Sci Med 2014;120:378–86. 10.1016/j.socscimed.2014.02.019 24666656

[35] Ndima SD, Sidat M, Give C Supervision of community health workers in Mozambique: a qualitative study of factors influencing motivation and programme implementation. Hum Resour Health 2015;13:63 10.1186/s12960-015-0063-x 26323970 PMC4556309

[36] Roome E, Raven J, Martineau T Human resource management in post-conflict health systems: review of research and knowledge gaps. Confl Health 2014;8:18 10.1186/1752-1505-8-18 25295071 PMC4187016

[37] Rowe AK, de Savigny D, Lanata CF How can we achieve and maintain high-quality performance of health workers in low-resource settings? Lancet 2005;366:1026–35. 10.1016/S0140-6736(05)67028-6 16168785

[38] Prashanth N, Marchal B, Hoeree T How does capacity building of health managers work? A realist evaluation study protocol. BMJ Open 2012;2:e000882 10.1136/bmjopen-2012-000882 PMC3330260 22466036

[39] Najafizada SA, Labonté R, Bourgeault IL Community health workers of Afghanistan: a qualitative study of a national program. Confl Health 2014;8:26 10.1186/1752-1505-8-26 25904976 PMC4405840

[40] Namakula J, Witter S Living through conflict and post-conflict: experiences of health workers in northern Uganda and lessons for people-centred health systems. Health Policy Plan 2014;29(Suppl 2):ii6–14. 10.1093/heapol/czu022 25274642 PMC4202915

[41] Kok MC, Kane SS, Tulloch O How does context influence performance of community health workers in low- and middle-income countries? Evidence from the literature. Health Res Policy Syst 2015;13:13 10.1186/s12961-015-0001-3 25890229 PMC4358881

[42] Brazier E, Fiorentino R, Barry MS The value of building health promotion capacities within communities: evidence from a maternal health intervention in Guinea. Health Policy Plan 2015;30:885–94. 10.1093/heapol/czu089 25148842 PMC4524340

[43] Dooris M, Poland B, Kolbe L Healthy settings: building evidence for the effectiveness of whole system health promotion—challenges and future directions. In: McQueen DV, Jones CM, eds. Global perspectives on health promotion effectiveness. New York, NY: Springer, 2007:327–52.

[44] McCoy DC, Hall JA, Ridge M A systematic review of the literature for evidence on health facility committees in low- and middle-income countries. Health Policy Plan 2012;27:449–66. 10.1093/heapol/czr077 22155589

[45] Kazi MAF Realist Evaluation for Practice. Br J Soc Work 2003;33:803–18. 10.1093/bjsw/33.6.803

[46] Marchal B, Dedzo M, Kegels G Turning around an ailing district hospital: a realist evaluation of strategic changes at Ho Municipal Hospital (Ghana). BMC Public Health 2010;10:787 10.1186/1471-2458-10-787 21184678 PMC3019197

[47] Van Belle SB, Marchal B, Dubourg D How to develop a theory-driven evaluation design? Lessons learned from an adolescent sexual and reproductive health programme in West Africa. BMC Public Health 2010;10:741 10.1186/1471-2458-10-741 21118510 PMC3001738

[48] Pawson R, Tilley N Realistic evaluation. London: Sage, 1997.

[49] Merton RK Social theory and social structure. New York: The Free Press, 1968.

[50] Pawson R, Tilley N Realist Evaluation. http://www.communitymatters.com.au/RE_chapter.pdf2004

[51] Koening G Realistic evaluation and case studies: stretching the potential. Evaluation 2009;15:9–30. 10.1177/1356389008097869

[52] Pawson R, Sridharan S Theory-driven evaluation of public health programmes. In: Killoran A, Kelly MP, eds. Evidence-based public health: effectiveness and efficiency. Oxford, UK: Oxford University Press, 2010:49.

[53] Deci EL, Ryan RM Cognitive evaluation theory. In: Deci EL, Ryan RM, eds. Intrinsic motivation and self-determination in human behavior. New York: Springer USA, 1985:43–112.

[54] Freudenberger HJ Staff burnout. J Soc Issues 1974;30:159–65. 10.1111/j.1540-4560.1974.tb00706.x

[55] Campbell JP, Dunnette MD, Lawler EE Managerial behaviour, performance, and effectiveness. New York: McGraw-Hill, 1970.

[56] Meyer JP, Allen NJ A three-component conceptualization of organizational commitment. Hum Resour Manag Rev 1991;1:62–89. 10.1016/1053-4822(91)90011-Z

[57] Greenberg J A taxonomy of organizational justice theories. Acad Manag Rev 1987;12:9–22.

[58] Yin R Case study research: design and methods. 3rd edn London: Sage Publications, 2003.

[59] International Guidelines for Test Use. International Journal of Testing 2001 https://www.intestcom.org/files/guideline_test_use.pdf (accessed August 2016).

[60] Ritchie J, Lewis J, eds. Qualitative research practice: a guide for social science students and researchers. London: SAGE Publications, 2003.

[61] Hayes A Introduction to mediation, moderation, and conditional process analysis: a regression-based approach. New York: The Guilford Press, 2013.

[62] Dalkin SM, Greenhalgh J, Jones D What's in a mechanism? Development of a key concept in realist evaluation. Implement Sci 2015;10:49 10.1186/s13012-015-0237-x 25885787 PMC4408605

[63] Adams A, Sedalia S, McNab S Lessons learned in using realist evaluation to assess maternal and newborn health programming in rural Bangladesh. Health Policy Plan 2016;31:267–75. 10.1093/heapol/czv053 26104820

[64] Strachan DL, Källander K, Nakirunda M Using theory and formative research to design interventions to improve community health worker motivation, retention and performance in Mozambique and Uganda. Hum Resour Health 2015;13:25 10.1186/s12960-015-0020-8 25925007 PMC4426548

[65] Dieleman M, Gerretsen B, van der Wilt GJ Human resource management interventions to improve health workers’ performance in low and middle income countries: a realist review. Health Res Policy Syst 2009;7:7 10.1186/1478-4505-7-7 19374734 PMC2672945

